# Protective Effect of a Polyherbal Traditional Formula Consisting of *Rosa damascena* Mill.,* Glycyrrhiza glabra* L. And* Nardostachys jatamansi *DC., Against Ethanol-induced Gastric Ulcer

**Published:** 2017

**Authors:** Zahra Memariani, Mannan Hajimahmoodi, Bagher Minaee, Fariba Khodagholi, Asal Yans, Roja Rahimi, Gholamreza Amin, Ghazaleh Moghaddam, Tayebeh Toliyat, Mohammad Sharifzadeh

**Affiliations:** a *Department of Traditional Pharmacy, Faculty of Traditional Medicine, Tehran University of Medical Sciences, Tehran, Iran. *; b *Department of Drug and Food Control, Faculty of Pharmacy, Tehran University of Medical Sciences, Tehran, Iran.*; c *Persian Medicine and Pharmacy Research Center, Tehran University of Medical Sciences, Tehran, Iran.*; d *Department of Histology, School of Medicine, Tehran University of Medical Sciences, Tehran, Iran. *; e *Neuroscience Research Center, Shahid Beheshti University of Medical Sciences, Tehran, Iran. *; f *Department of pharmacognosy, faculty of pharmacy, Tehran University of Medical Sciences, Tehran, Iran.*; g *Deptartment of Pharmaceutics, Faculty of Pharmacy, Tehran University of Medical Science, Tehran, Iran.*; h *Department of Pharmacology and Toxicology, Faculty of Pharmacy, and Pharmaceutical Sciences Research Center, Tehran University of Medical Sciences, Tehran, Iran.*

**Keywords:** Antioxidant, *Glycyrrhiza glabra*, Heme-oxygenase-1, Inflammation, *Nardostachys jatamansi*, Peptic ulcer, *Rosa damascena*

## Abstract

“VARD” formula consisting of* Rosa damascena *Mill. (Rosaceae) petals, and rhizomes of *Glycyrrhiza glabra* L. (Papilionaceae) and* Nardostachys jatamansi* DC. (Valerianaceae), has been proposed for gastric ulcer in Iranian traditional medicine. We investigated the antiulcer activity of each plant separately and in combination. The biochemical and molecular functions of extracts were also evaluated. Each plant hydroalcoholic extract was standardized via determination of total phenolic and flavonoid contents, also via some phenolic compounds determination and specially glycyrrhizic acid in *G. glabra *by using HPLC. Rats received orally extracts of the plants (20, 40 and 80 mg/Kg) and “VARD” (45 mg/Kg) 1 h before ethanol administration. Two h after receiving ethanol, animals were sacrificed; the stomach was removed for macroscopic and microscopic assessment. Also heme-oxygenase-1, glutathione, and catalase were measured in the gastric tissue of the rats pretreated by “VARD” and dose of 20 mg/Kg of extracts. Among three extracts,* R. damascena *and *G. glabra* contained more total phenolic and flavonoid content respectively. Gallic acid was prominent compound in *R. damascena.* The extracts of *R. damascena*, *G. glabra*, and *N. jatamansi* significantly decreased ulcer index. ED_50_ values were 8.2, 31.86 and 25.08 mg/Kg respectively. “VARD” significantly decreased ulcer index compared to 20 mg/Kg of *G. glabra* (*p *< 0.0001) and* N. jatamansi* (*p *< 0.001). Pretreatment with “VARD” and each plant extracts (20 mg/Kg) increased glutathione, catalse and heme-oxygenase-1 significantly (*p *< 0.05**) **in comparison with control group. Our findings indicate that “VARD” partly via antioxidant activity can be considered as an effective antiulcer formula.

## Introduction

Peptic ulcer regarded as a relatively common gastrointestinal disorder which can affect individuals during their lives with risk of developing complications, such as hemorrhage, perforation, penetration, obstruction of the gastric outlet, and the consequent mortality (1, 2). Gastric ulcer is disruption in the continuity of the stomach mucosa. It may be associated with imbalance between the mucosal protective and invasive factors (3, 4). Oxidative stress is known to be as one of the major aspects in the pathophysiology of the gastric diseases. Parameters including psychological and physical stress, microbial infection, and ethanol overexposure induce oxidative stress in the stomach (5). Ethanol overexposure leads to generation of the reactive oxygen species (ROS), such as hydrogen peroxide, superoxide anion, and hydroxyl radical that are the causative factors for mucosal lesions via oxidative damage in ethanol-induced gastric ulcers (6). Glutathione and catalase as two essential endogenous antioxidant agents can establish one of the most important cytoprotective mechanisms against lesion formation (7, 8). Moreover, upregulation of the heme-oxygenase-1 (HO-1), a stress response protein, is associated with antioxidant and anti-inflammatory activity (9, 10). In Iranian traditional medicine (ITM), there are so many medicinal plants for prevention and treatment of gastrointestinal disorders such as peptic ulcer (11). A multi-herbal formula traditionally named “VARD” is mentioned in several important ITM literatures such as Canon of medicine (Avicenna) (11), Zakhireh-kharazmshahi (Jorjani) (12), Kholasat-ul-hekma (Aqili khorasani) (13), Exir-e-azam (Chishti) (14) and Hidayat-al-Mutaallimin fi-al-Tibb (al-Akawayni al-Bokhari) (15), for the treatment of gastric disorders. “Vard-e-Ahmar” (*Rosa damascena* Mill*.* [Rosaceae])*, *“Soos” (*Glycyrrhiza glabra *L*. *[Papilionaceae])*,*and “Sonbol-e-tib”(*Nardostachys jatamansi* DC*.*[Valerianaceae]) are the ingredients of this formula (11-15). The antioxidant properties of the *G. glabra* (16), *R. damascena *(17) and *N. jatamansi* (18, 19) have been previously investigated and found to possess free radical scavenging properties *in-vitro*. Some of their chemical compounds have also been appeared to induce significant antioxidant activity and increase the levels of endogenous antioxidant enzymes (20-23). The antiulcer properties of *G. glabra* have been mentioned in numerous reports (24).To date there is not any investigation addressing the antiulcer activity of the *R. damascena* and *N. jatamansi* and the traditional polyherbal formula “VARD” thus far. In the present study, we aimed to determine some phenolic compounds in the extracts of *R. damascena*, *G. glabra* and *N. jatamansi* and additionally glycyrrhizic acid in *G. glabra* by HPLC method as well as total phenolic and flavonoid contents, and evaluate the pharmacological, histological, and biochemical properties of the plants extracts and “VARD” against ethanol-induced gastric ulcer in rats.

## Materials and methods

*Materials*

All the chemicals were analytical grade and purchased from Merck (Darmstadt, Germany). Antibodies directed against HO-1 and *β*-actin were obtained from Cell Signaling Technology (Danvers/MA, USA).

*Plant material and extract preparation*

Rose dried petals and dried rhizomes of the licorice and jatamansi were purchased in April (2013) from local herbal store of Tehran and authenticated by one of authors (G. Amin), and voucher specimens (*Rosa damascena* Mill. [Rosaceae; No: PMP-507], *Glycyrrhiza glabra* L. Var glabra [Papilionaceae; No: PMP-221] and *Nardostachys jatamansi* DC. [Valerianaceae; No:PMP-220] were deposited in the herbarium of faculty of pharmacy, Tehran University of Medical Sciences. Dried sample of each plant (150 g) was extracted with ethanol (70%) at room temperature for 4 days. Each extracted solution was filtered and evaporated to dryness at 40 °C to yield residues about 19.65, 17.86, and 14.67 % on the basis of dried plant material for *R. damascena*, *G. glabra,* and *N. jatamansi* respectively.

VARD formula was prepared from the dried extracts of *R. damascena, G. glabra, *and* N. jatamansi* respectively by the ratio of 2:1:1 to the dose of 45 mg/Kg which contains 22.5 mg/Kg, 11.25 mg/Kg and 11.25 mg/Kg of *R. damascena, G. glabra, *and* N. jatamansi* respectively in it. 

*Determination of total phenolic content*

Total phenolics of each sample were determined using folin-ciocalteu reagent (25). Each prepared sample (1 mL) was mixed with folin-ciocalteu reagent (1.5 mL) which previously diluted 10-fold with distilled water, and allowed to stand at room temperature for 5 min. 1.5 mL of bicarbonate solution (60 g/L) was added to the mixture. After incubation for 90 min at room temperature, the absorbance was measured at 725 nm using a UV-visible spectrophotometer (GBC, Cintra 40). Total phenolics were quantified by calibration curve obtained from measuring the absorbance of the known concentrations of gallic acid standard solutions. All tests were carried out in triplicate and the results were expressed as gallic acid equivalents (mg GAE/g dry weight).

*Determination of total flavonoid content*

Total flavonoid content was determined by the aluminum chloride colorimetric method (26). Concisely, 1 mL of each prepared sample was added to 10 mL volumetric flask containing 4 mL of double distilled water. 0.3 mL NaNO_2 _(5%) was added to the flask and 5 min later 0.3 mL AlCl_3_ (10%) was added. After 6 min, 2 mL NaOH (1 M) was added and the total volume was made up to 10 mL and the flask contents were thoroughly mixed*. *The absorbance level was measured versus blank at 510 nm (GBC, Cintra 40). Total flavonoid contents were represented as mg catechin equivalents (CE) per one gram dry extract according to the catechin standard solutions. 

*High performance liquid chromatographic analysis*

Each sample was analyzed by Knauer HPLC (Germany) system consisting of a pump (Maxi-Star K-1000, Knauer, Germany), a degasser, an automated injector, a column oven, and aUV detector. The system was controlled by EuroChrom 2000 software (Version 1.6, Knauer Co., Germany). For determination of the phenolic compounds in samples, chromatographic conditions were evaluated and optimized in Eurospher-100 C18 column (5 μM, 4.6 × 250 mm). Column temperature was maintained at 30 °C. Mobile phase consisting of methanol (A) and acetic acid in water (B) (3:97 v/v), and the flow rate was set at 1 mL/min. The chromatographic detection was monitored at 280 nm. 

*Glycyrrhizic acid determination in G. glabra*

The detection of glycyrrhizic acid in *G. glabra* was also carried out using another HPLC method (27). Column temperature was maintained at 25 °C. Mobile phase consisting of acetic acid, acetonitril, H_2_O (1:38:68 v/v), and the flow rate was set at 1 mL/min. The chromatographic detection was monitored at 254 nm. The injection volume for all samples was 10 μL. Compounds were identified according to the retention times as a comparison with the corresponding standards. The concentration of each compound was measured from peak area based on calibration curves. All amounts were expressed as milligram per gram of the dry extracts (mg/g).

*Animals*

Wistar male rats weighting between 180 and 220 g were used in this study. Animals were left under standard conditions (23 ± 2 °C, 12 light-dark cycles) and had free access to water and standard pellet diet. Food was withdrawn 24 h before experiments, though they had free access to water. For each group, seven rats were used. Animal experiments have been carried out under standard condition according to the ethical guidelines of committee of Tehran University of Medical Sciences for animal study. 

*Gastric ulcer induction by ethanol*

Rats were randomly divided into 12 groups comprising seven individuals in each group. Following 24 h fasting period, animals were orally received distilled water (1mL/200 g of body weight) as control group, ranitidine (50 mg/Kg), plant extracts at different doses (20, 40 and 80 mg/Kg), or VARD (45mg/Kg) in separate groups. Subsequent to 1 h all groups received ethanol 80% (1 mL/200 g of body weight). Following 2 h after ethanol administration, animals were sacrificed, stomachs were removed and rinsed with normal saline (NaCl 0.9%), and cut along great curvature speared on a flat surface and examined macroscopic for ulceration scoring (28).

*Measurement of ulcer index and calculation of protection percentage*

Gastric ulcers were measured macroscopically and scored with an arbitrary system:

Score 1: each fifth petechia was calculated as 1 mm 

Score 2: lesion length between 1 and 2 mm 

Score 3: lesion length between 2 and 4 mm 

Score 4: lesion length between 4 and 6 mm 

Score 5: lesion length more than 6 mm

The ulcer index (UI) was calculated in each animal group by the following formula (28):

$\mathrm{UI}=\sum_{i=1}^{5}i\times n_{i}$

(1)

Where, i is score number and n_i_ is number of ulcers of score *i*th.

The protection percentage was calculated using the following formula (28):

$\mathrm{Protection percentage}=\frac{{\mathrm{UI}}_{c}-{\mathrm{UI}}_{t}}{{\mathrm{UI}}_{c}}\times 100$

(2)

Where, UI_c_ and UI_t_ are the ulcer indexes of control and test groups, respectively.

*Histological survey*

For microscopic examination, gastric tissues were fixed in freshly made 10% formaldehyde and processing and sectioning steps were conducted afterwards. Microscopic slides were stained by hematoxylin–eosin methods. Finally, sections were analyzed using a light microscope (29).

*Determination of glutathione levels*

Reduced glutathione (GSH) levels were determined using dithionitrobenzoic acid (DTNB) method in whole cell lysate of the gastric tissue at 412 nm (30).

*Catalase activity assay*

Catalase (CAT) activity was assessed by Aebi method (31). Briefly, tissue lysate (200 μL) was added to a cuvette containing 50 mM phosphate buffer (pH 7.0); subsequently, 1 mL of freshly prepared 30 mM H_2_O_2_ was added to start the reaction. The rate of the substrate (H_2_O_2_) decomposition was further evaluated at 240 nm. 

*Western blot analysis*

Gastric tissues were harvested into lysis buffer containing complete protease inhibitor cocktail. Protein concentrations were determined according to the Bradford’s method (32). Electrophoresis of the total proteins was conducted in 12.5% SDS-PAGE gels, and then proteins were transferred on polyvinylidene fluoride membranes, probed with specific HO-1 and secondary antibodies. Immunoreactive polypeptides were detected by chemiluminescence using enhanced ECL reagent (Amersham Bioscience, USA) and autoradiography. By densitometric scan of films, the results were quantified. Data analysis was done by ImageJ, via measuring integrated density of bands in triplicate after background subtraction. 

*Statistical analysis*

Results were expressed as mean ± SEM. The statistical difference between each two determined groups was calculated by using one-way ANOVA and new man-keuls multiple comparison post hoc tests. The significance analysis was performed using the Graphpad Prism 5.0.

## Results

*Determination of total phenolic and total flavonoid content*

The total phenolic content of *R. damascena*, *G. glabra* and *N. jatamansi* extracts, was 217.728 ± 0.13,15.792 ± 0.26, and 11.616 ± 0.21 mg GAE/g respectively. The total flavonoid content of each extract was 22.8 ± 0.18, 126.36 ± 0.41 and 35.28 ± 0.23 mg of CE/g of dry extract respectively by reference to the related standard curves.

**Table 1:** Phenolic compounds HPLC analysis of the *R. damascena*, *G. glabra* and *N. jatamansi* hydroalcoholic extracts

**Phenolic compound**	**Plant extract**		
	***R.damascena***	***G. glabra***	***N. jatamansi***
**Gallic acid**	118.213 ± 0.12	-	-
**Caffeic acid**	-	0.95 ± 0.13	-
**Syringic acid**	3.48 ± 0.19	-	2.78 ± 0.17
***ρ*** **-coumaric acid**	-	5.71 ± 0.23	-
**Ferulic acid**	-	5.93 ± 0.2	-
**Quercetin**	12.86 ± 0.31	-	-

Note: Each value represents the mean ± SEM (n = 3). The amount of compounds was expressed as mg/g in dry extract.

**Figure 1:**
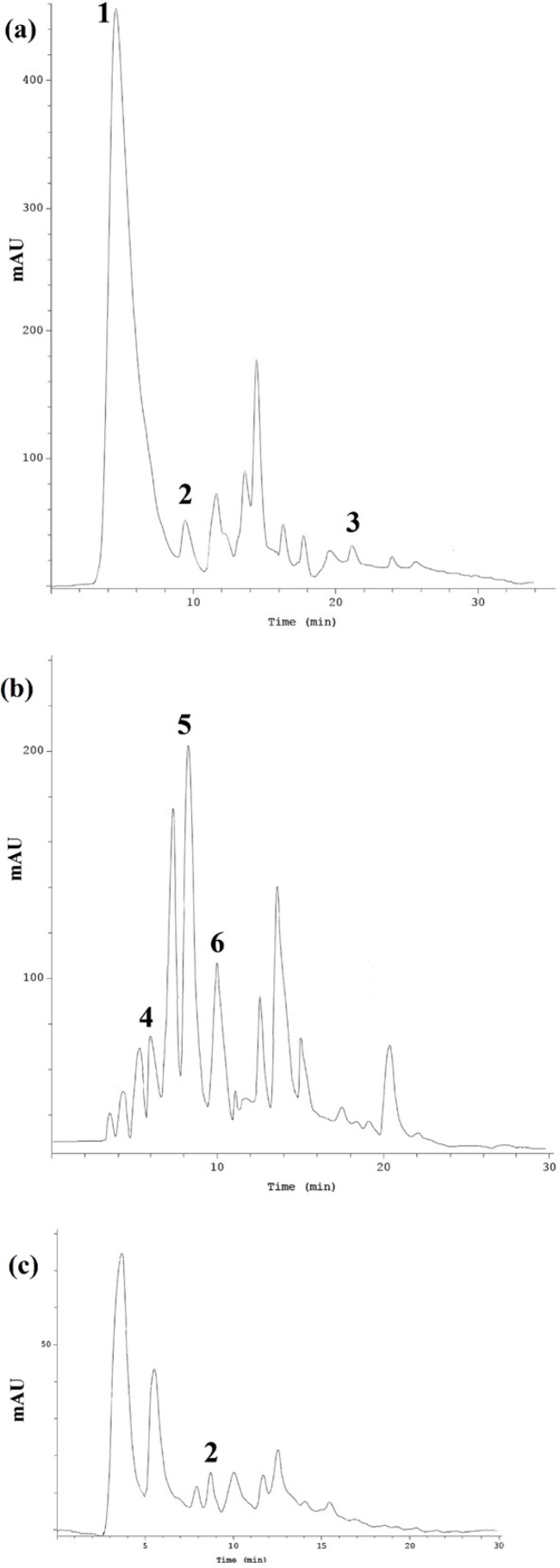
HPLC chromatogram of phenolic compounds 1: Gallic acid, 2: Syringic acid, 3: Quercetin, 4: Caffeic acid, 5: p-Cumaric acid, 6: Ferulic acid detected in RDHE (a), GGHE (b) and NJHE (c) hydroalcoholic extracts

**Figure 2.**
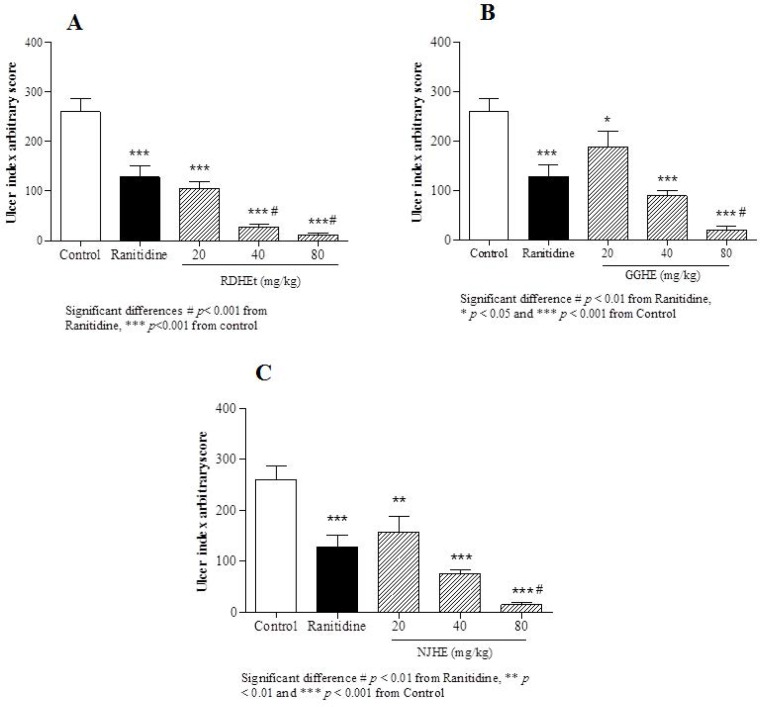
Gastric ulcer index induced by ethanol in animal groups pre-treated by RDHE (A), GGHE (B) and NJHE (C). The results are expressed as mean ± SEM. (n = 7). The animals received: vehicle (water), ranitidine (50mg/kg), and doses of plant extract (20, 40 and 80 mg/kg, respectively). Statistical comparison was performed using analysis of variance (ANOVA) followed by post hoc Newman keuls test (* *p *< 0.05, ** *p *< 0.01 and *** *p *< 0.001

**Figure 3:**
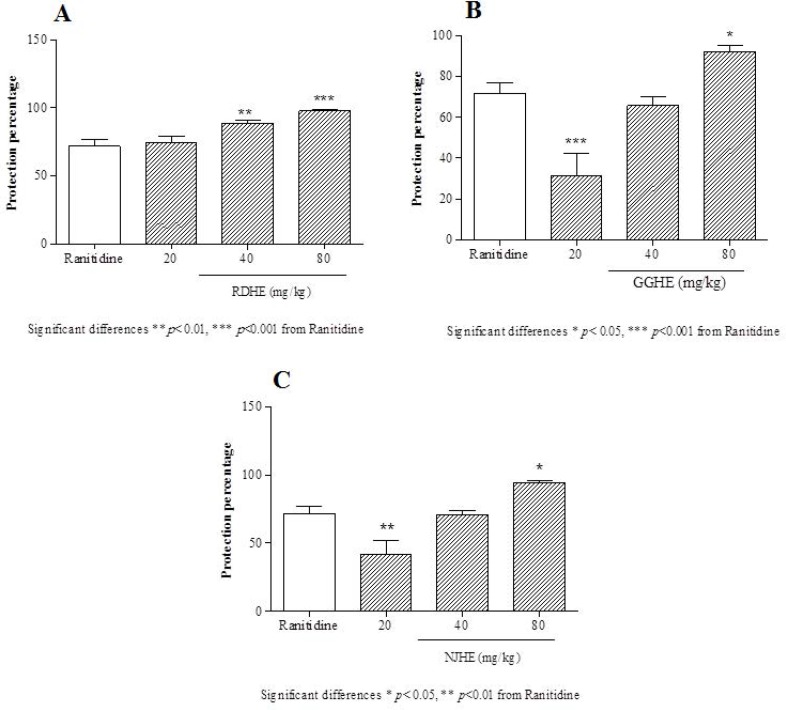
Protective effects of the RDHE (A), GGHE (B) and NJHE (C) against gastric lesions induced by ethanol. The results are expressed as mean ± SEM. (n = 7). The animals received ranitidine (50 mg/kg), and doses of plant extract (20, 40 and 80 mg/kg, respectively). Statistical comparison was performed using analysis of variance (ANOVA) followed by post hoc Newman keuls test (* *p *< 0.05, ** *p *< 0.01 and *** *p *< 0.001

**Figure 4:**
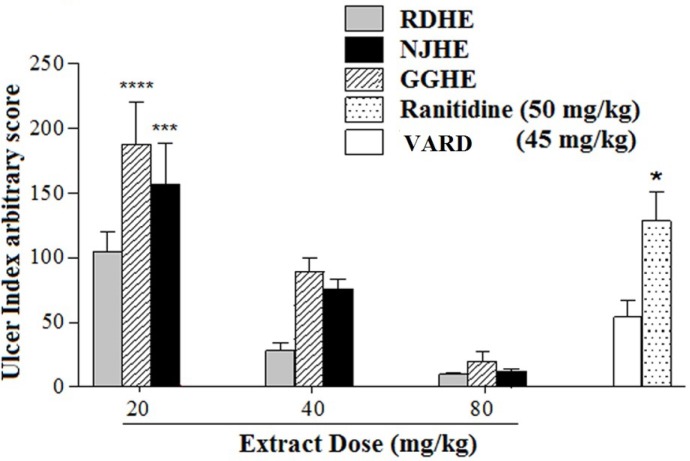
Ulcer index in animal group pre-treated by VARD formula compared with all other groups. Statistical comparison was performed using analysis of variance (ANOVA) followed by post hoc bonferroni test. Difference from control group: * *p *< 0.05, *** *p *< 0.001 and **** *p *< 0.0001

**Figure 5:**
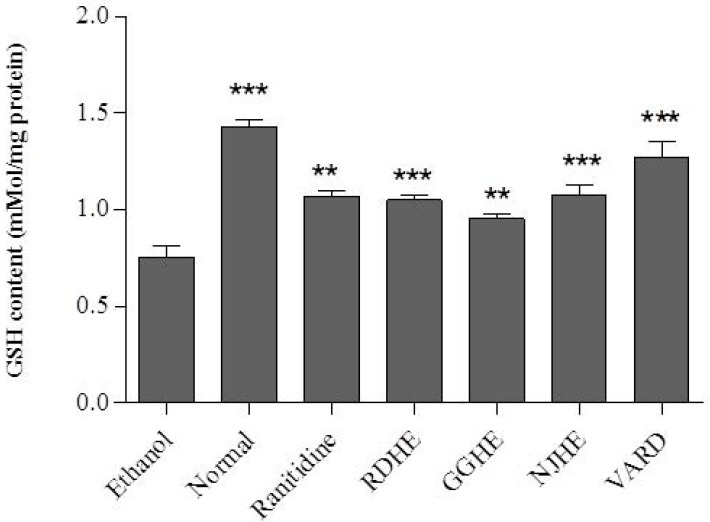
GSH content of rat gastric tissues in RDHE, NJHE, GGHE (20 mg/kg), VARD (45 mg/kg) and ranitidine (50 mg/kg). ** *p *< 0.01 and *** *p *< 0.001 significantly different from the control

**Figure 6:**
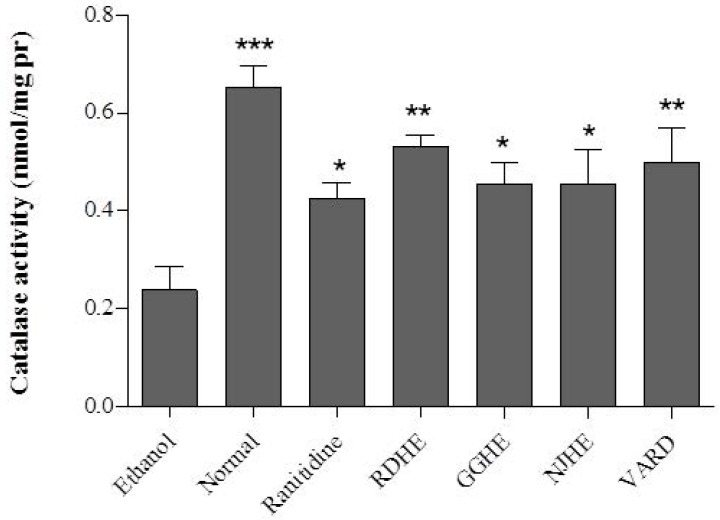
CAT activity in rat gastric tissues in RDHE, NJHE, GGHE (20 mg/kg), VARD (45 mg/kg) and ranitidine (50 mg/kg). * *p *< 0.05, ** *p *< 0.01 and *** *p *< 0.001 significantly different from the control

**Figure 7:**
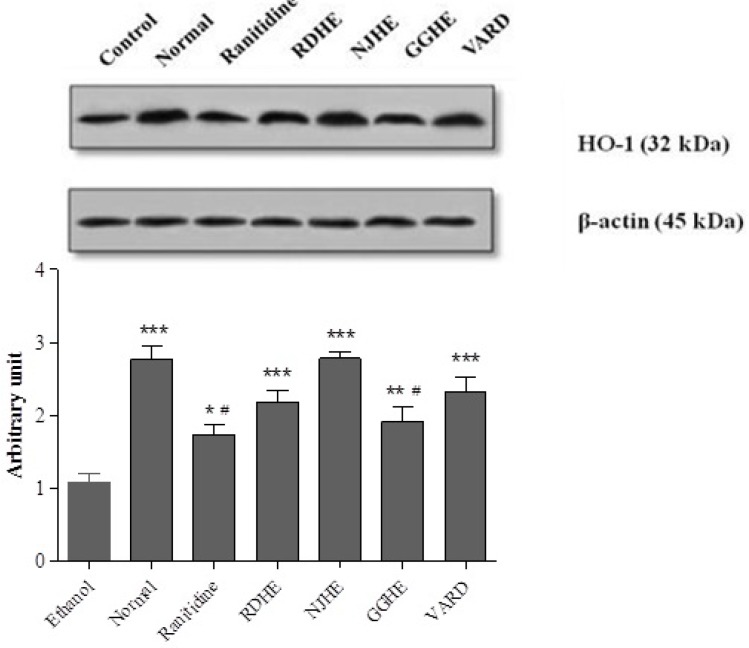
Western blot analysis of RDHE, NJHE, GGHE (20 mg/kg), VARD (45 mg/kg) and ranitidine (50 mg/kg) effects on HO-1 levels in gastric tissue. Proteins separation was conducted on SDS-PAGE, and then proteins blotted, probed with anti-HO-1 antibody and reprobed with anti-β-actin antibody. The densities of HO-1 bands on emerged films were measured and the ratio calculated. *** *p *< 0.001 significantly different from the control. # *p *< 0.05 significantly different from NJHE

*HPLC analysis of the extracts*

Results of HPLC determination of phenolic compounds in each extract have been represented in Table 1. Phenolic compounds like phenolic acids, flavonoids and polyphenols, and also glycyrrhizic acid are known to be as the important components of the formula intended for gastric ulcer treatment. HPLC method was applied to evaluate five phenolic acids including gallic acid, caffeic acid, *ρ*-coumaric acid, ferulic acid, and syringic acid and quercetin in *R. damascena* hydroalcoholic extract (RDHE), *G. glabra* hydroalcoholic extract (GGHE) and *N. jatamansi* hydroalcoholic extract (NJHE). Figure 1. depicts the chromatogram of phenolic compound in RDHE (a), GGHE (b), and NJHE (c). Data showed that the amount of gallic acid is considerable in RDHE (118.213 ± 0.12 mg/g), while it was not detected in other two plants. The quercetin amount, as an important flavonoid in *R. damascena* was 12.86 ± 0.31 mg/g.

As mentioned before, for detection of the glycyrrhizic acid in GGHE, another HPLC method was used, and as result the amount of glycyrrhizic acid in GGHE was 27.12 ± 0.13 mg/g. Among five phenolic compounds, only syringic acid was detected in NJHE (2.785 ± 0.17 mg/g) (Table 1.).

*Measurement of ulcer index and calculation of protection percentage*

Ethanol-induced gastric ulcer model was used in this study. Based on the present study, in the ethanol-induced ulcer protocol, administration of ethanol 80% led to apparent gastric ulcers in rats associated with marked increase UI (Figure 2.). In macroscopic examination of the stomachs, oral administration of three doses (20, 40 and 80 mg/Kg) of RDHE, GGHE, and NJHE caused a notable inhibition of ethanol-induced gastric ulcer as compared to the control group.

The effective dose 50 (ED_50_) values were 8.2, 31.86, and 25.08 mg/Kg respectively for RDHE, GGHE, and NJHE. Administration of doses 40 and 80 mg/Kg of RDHE (*p *< 0.001), and dose 80 mg/Kg of both GGHE and NJHE (*p *< 0.01) provoked a remarkable decrease in the UI compared to the ranitidine (50 mg/Kg) group (Figure 2.). Moreover doses 40 and 80 mg/Kg of RDHE (*p *< 0.01 and *p *< 0.001), and dose 80 mg/Kg of both GGHE and NJHE (*p *< 0.05) significantly induced more protection percentage in comparison with ranitidine (Figure 3.). Figure 4. Shows ulcer inhibition of the VARD in ethanol-induced gastric ulcer compared with all other groups. As it has been indicated, VARD significantly had more gastroprotective effect than GGHE 20 mg/Kg (*p *< 0.0001), NJHE 20 mg/Kg (*p *< 0.001), and ranitidine 50 mg/Kg (*p *< 0.05).

*Histological examination*

In the control group severe mucosal damage was appeared as hemorrhagic lesions. The submucosa was edematous and inflammatory reaction and bleeding was observed. Ranitidine pretreated rats showed normal mucosa and generally mild inflamed tissue without ulceration. On the other hand, animals pretreated with plants hydroalcoholic extracts showed mild mucosal damage and submucosal edema. In RDHE (20mg/Kg), GGHE (20mg/Kg) and NJHE (20 mg/Kg) pretreated groups, submucosa had a mild inflammation, mucosal layer was thin with no or little ulceration and bleeding. By increasing the dose of each extract, the severity of the ulcers and inflammation of submucosa were reduced in microscopic evaluation. Administration of VARD (45 mg/Kg) caused an apparent decrease in edema along with normal gastric tissue and mucosal layer.

*Determination of GSH level and CAT activity*

Oral administration of ethanol 80% significantly led to decreased GSH level (*p *< 0.001) and CAT activity (*p *< 0.01) in gastric tissue of the control group compared with normal rat (Figures 5. and 6.). Pretreatment of rats with 20 mg/Kg of each plant extracts (RDHE, GGHE, and NJHE), ranitidine (50 mg/Kg), and VARD significantly increased GSH content in gastric tissue when compared with control group (*p *< 0.001 and *p *< 0.01; Figure 5.). All the extracts at dose 20 mg/Kg, ranitidine (50 mg/Kg) and VARD (45 mg/Kg) were capable of increasing the activity of CAT in gastric tissue as compared by control (Figure 6.). However there was not any considerable difference in CAT and GSH levels between each two extracts group (*p*< 0.05).

*Measurement of HO-1 protein level*

In order to investigate the effect of pretreatment with extracts (1 h) prior to ethanol 80% exposure on HO-1 induction, the protein level of cell lysates was determined by western blot assay. Figure 7. represents that pretreatment with 20 mg/Kg of these extracts increased HO-1 levels compared to control group. In addition a significant increase in HO-1 level was also occurred in groups received VARD (*p *< 0.001) and ranitidine (*p *< 0.05) compared with the control group. Interestingly, NJHE could dramatically elevate HO-1 level more than ranitidine (*p *< 0.01) and GGHE (*p *< 0.05).In conclusion, it can be inferred that regardless of ranitidine (*p *< 0.01) and GGHE (*p *< 0.01) groups, there was not any significant difference in HO-1 level between test groups and normal rats.

## Discussion

In the current study, we have provided evidence that oral administration of the hydroalcoholic extracts of the “VARD” formula and its herbal ingredients including *R. damascena*, *N. jatamansi* and *G. glabra* have protective effect against gastric lesions induced by ethanol in rats. These protective properties were interestingly comparable with 50 mg/Kg of ranitidine. The polyherbal “VARD” formula had significantly more protective effect than ranitidine and each ingredient, GGHE (20 mg/Kg), and NJHE (20 mg/Kg). *R. damascena* which is the main compound of the formula has considerable antioxidant activity due to phenolic composition (33). For *R. damascena* extract the amount of total phenol content have been reported as 276.02 ± 2.93 mg GAE/g (33), which is similar to our results (217.728 ± 0.13 mg GAE/g). Additionally the amount of gallic acid (118.213 ± 0.12 mg/g) in RDHE was also about 54% of total phenol content. Vinokur *et al*. (2006) has previously found that the levels of gallic acid in some samples of teas prepared from rose petals are 35-55% of the total phenol content (34). Considering the notable contents of total phenol and flavonoids, significant antioxidant activities have been shown by these natural compounds (21, 35). Various studies have repeatedly indicated the antiulcer activities of *G. glabra *(24). Although major compounds such as glycyrrhizinic acid and its derivative carbenoxolone may have antiulcer properties through promotion mucous secretion and cell proliferation in the stomach, high flavonoid content could also be crucial in terms of gastroprotective property via potent antioxidant capability (24, 36 and 37). Total flavonoid content in GGHE (126.36 ± 0.41mg CE/g of dry extract) is comparable with that amount (91.75 ± 6.61) reported by Dong *et al*. (2014) (38). In another study total flavonoid content in flavonoid-rich fraction of *G. glabra* has been shown as 262.8 mg quercetin equivalents/g (39).

Alongside this issue, due to high quantity of *R. damascena* in this formula, gastroprotective action of “VARD” might be attributed to its high phenolic compounds which provide antioxidant and antiulcer activity to such compounds (40). Another ingredient of “VARD”, *N. jatamansi,* has also remarkable antioxidant and free radical scavenging properties (41) that can be ascribed to its polyphenolic substances (42). Although *N. jatamansi* and *R. damascena* can be used in gastrointestinal disorders (11) there is no evidence concerning their antiulcer function. This study represents the protective effect of both NJHE and RDHE against ethanol induced gastric ulcer in rat. From the macroscopic and microscopic examinations, all pretreated groups showed significant reduction of hemorrhagic lesion area in gastric mucosa compared with the ethanol group. Ethanol, as an oxidative stress agent, brings about proceeding cell death in gastric mucosal cells and peptic ulcers. Mucosal damage leads to generate reactive oxygen and free radicals. In experimental animals and humans the damage is associated with a significant decrease in GSH and CAT values in gastric tissue (43-45). Due to intracellular antioxidants such as GSH and CAT, the ability of cells to resist against oxidative stress is anticipated to be increased. While it has been appeared a significant decrease in both GSH and CAT levels in ethanol group, these parameters were significantly increased following pretreatment of animals with RDHE, GGHE, NJHE, and VARD. Several lines of evidences have indicated the inducing effect of phenolic compounds on endogenous antioxidant factors (21-23). Yeh *et al*. (2009) reported that gallic acid, ferulic acid and *p*-coumaric acid significantly increased the GSH content and augmented the activity of antioxidant enzymes, such as CAT in rat cardiac cells (23). Gallic acid with strong antioxidant properties (20) has also been known to exert significant recovering effect on GSH reduction induced by CCl_4_ in rat hepatocytes (46). In the cellular antioxidative defense system, up-regulation of HO-1 is an adaptive response for increase of cell resistance to oxidative stress (47). Growing evidences have delineated that some natural compounds can defend cells against oxidative injury through HO-1 induction (22, 48-50). Bae *et al*. (2012) showed that a biologically active fraction of *N. jatamansi* decreases the severity of pancreatitis via HO-1 induction in mice (51). Quercetin has been shown to have protective effect via HO-1 induction against ethanol-derived oxidative stress in human hepatocytes (22) and H_2_O_2_-induced apoptosis in macrophages (52). Glycyrrhetic acid, the derivative of glycyrrhizin from *G. glabra* demonstrated protective role in carbon tetrachloride (CCl_4_)-induced liver injury via increasing HO-1 (21). Furthermore, some evidence indicated that glycyrrhetic acid can affect the activity of cAMP-dependent protein kinase A (PKA) signal transduction which appears to be involved in many cellular and pathological conditions (53, 54). The effects of glycyrrhetic acid on the phosphorylation of some exact proteins by the kinase pathway may be related to its protective function in gastric ulcers. There is also body of evidence indicating that PKA activity can inhibit oxidative stress (55, 56). Recently, it has been demonstrated that activation of PKA regulated the cAMP-dependent HO-1 induction in rat hepatocyte culture (57, 58). Glycyrrhetic acid might have a protective role against oxidative stress and promote survival of gastric cells via interaction with both PKA and HO-1. 

However, according to these definitions, it can be deduced that the peptic ulcer prevention in rats established on glycyrrhetic acid were produced in part by interacting with PKA and subsequent neurotransmitter signaling pathways. In this experiment, we have demonstrated that pretreatment of the rats with GGHE, NJHE and RDHE or in combination by “VARD” formula could noticeably elevate the HO-1 value compared to the control group. Of further relevance, HO-1 induction represents another side of their antioxidant and anti-inflammatory properties (59). Various polyphenols have been reported to provide anti-inflammatory protection via induction of HO-1 (60) such as anti-inflammatory property of quercetin in mouse model of arteriosclerosis (61). HO-1 has been believed to offer anti-inflammatory effect and the consequent up-regulated HO-1 can decrease the expression of cyclooxygenase-2, inducible nitric oxide synthase (iNOS), tumor necrosis factor (TNF)-*α *and interleukin (IL)-6 as pro-inflammatory factors (62). Given these outcomes stated herein, the antioxidant property of phenolic compounds in “VARD” polyherbal formulation can modulate the level of oxidative stress which thereby protects from gastric ulcer formation by enhancing antioxidant or anti-inflammatory capacity. 

The combination of these plants not only have a significant therapeutic effect, but also can markedly diminish the possible side effects; which can cover the various reasons being involved in the pathogenesis of peptic ulcer such as psychological stress (63, 64). Lines of evidences represented that *R. damascena* and *N. jatamansi* have anti-depressant, hypnotic and anti-anxiety effects (65-68); which can suggest a reasonable justification for designing a safe and promising strategy against peptic ulcer according to these polyherbal traditional formulation.

## Conclusions

Collectively, our obtained results point toward a notion that “VARD” has significant protective effect against gastric ulcer. Furthermore, this study also designates that the gastroprotective activity of such compounds might be partly due to their antioxidant mechanisms. Other potential mechanisms persevere to be evaluated by study the effects of gallic acid, glycyrrhetic acid, quercetin or other compounds on PKA functions in our future investigation. More additional studies need to perform in order to elucidate further participating pathways being responsible for the antiulcer property of “VARD” formula. Also from the therapeutic standpoint, well-designed clinical trials are also suggested to investigate the protective and/or curative role of such desirable formula in peptic ulcer treatment.
